# Validation of a LC‐MS/MS Assay for Rapid and Simultaneous Quantification of Cobicistat and Venetoclax in Human Plasma and Serum

**DOI:** 10.1002/bmc.70179

**Published:** 2025-07-28

**Authors:** Niels Westra, Kai van Hateren, Jos G. W. Kosterink, Marjolijn N. Lub‐de Hooge, Thijs H. Oude Munnink, Daan J. Touw

**Affiliations:** ^1^ Department of Clinical Pharmacy and Pharmacology, University Medical Center Groningen University of Groningen Groningen The Netherlands; ^2^ PharmacoTherapy, Epidemiology & Economics, Groningen Research Institute of Pharmacy University of Groningen Groningen The Netherlands; ^3^ Pharmaceutical Analysis, Groningen Research Institute of Pharmacy University of Groningen Groningen The Netherlands

## Abstract

Measuring cobicistat and venetoclax concentrations in human plasma and serum facilitates therapeutic drug monitoring (TDM) and pharmacokinetic (PK) boosting studies. Therefore, the objective of this study was to develop and validate a rapid LC‐MS/MS analytical method for the simultaneous determination of cobicistat and venetoclax concentrations in plasma and serum. The method was validated according to EMA and FDA guidelines. Chromatographic separation was performed using a liquid chromatography (LC) system with a C18 column. The elution gradient involved two mobile phases: mobile phase A (ammonium formate) and mobile phase B (acetonitrile). The concentration range was 5–500 μg/L for cobicistat and 50–5000 μg/L for venetoclax. Accuracy and precision were within the required limits, with accuracy ranging from −5.9% to 2.4%, within‐day precision from 1.2% to 4.8%, and between‐day precision from 0.4% to 4.3%. Cobicistat and venetoclax were stable for at least 8 days under various storage and handling conditions. Clinical TDM samples showed mean concentrations ± standard deviation (SD) of 138.8 ± 123.3 μg/L for cobicistat and 1497.1 ± 1285.9 μg/L for venetoclax. The development and validation of this LC‐MS/MS assay provide a reliable and efficient method for the simultaneous quantification of cobicistat and venetoclax in plasma and serum samples.

## 1 Introduction

Venetoclax, an oral inhibitor of the anti‐apoptotic molecule B‐cell lymphoma (BCL‐2), has shown great efficacy in chronic lymphocytic leukemia (CLL) (Roberts et al. 2016; Seymour et al. 2018; Stilgenbauer et al. 2016). Furthermore, venetoclax–azacitidine is a favorable combination for first‐line treatment of unfit acute myeloid leukemia (AML) patients (DiNardo et al. 2020; Pratz et al. 2024). Despite its effectiveness in unfit AML patients, venetoclax–azacitidine is an expensive treatment regimen. Venetoclax is primarily metabolized by CYP3A4 and therefore a suitable candidate for pharmacokinetic (PK) boosting (Patel et al. 2021; Westra et al. 2023). The venetoclax dose can thereby be reduced, and the venetoclax–azacitidine combination is expected to be more cost‐effective in combination with a strong CYP3A4 inhibitor. Cobicistat is a strong irreversible CYP3A4 inhibitor and is primarily used to boost antiretroviral drugs (Deeks 2014). In the last decade, cobicistat has also been explored as a PK booster for several oncological drugs (Hohmann et al. 2021; Lubberman et al. 2017; Overbeek et al. 2023; van Veelen et al. 2022; Westra et al. 2023). The HOVON 171 trial (NCT06014489) aims to develop a venetoclax dosing strategy in combination with the strong irreversible CYP3A4 inhibitor cobicistat. For the HOVON 171 trial, the development and validation of an analytical method to quantify both cobicistat and venetoclax in serum and plasma was required.

Therapeutic drug monitoring (TDM) of cobicistat can be of guidance in specific cases (Hofman et al. 2023; Van Den Born‐Bondt et al. 2024). Furthermore, adherence monitoring of cobicistat can be a valuable factor when cobicistat is used as a PK booster for drugs to treat serious diseases (e.g., PK boosting of oncologic drugs). TDM of venetoclax is not recommended to guide the optimal efficacy of venetoclax (Mueller‐Schoell et al. 2021); however, TDM of venetoclax can be used for toxicity and interaction management (Kobayashi et al. 2022; Philippe et al. 2024; Wang et al. 2024). Several validated mass spectrometry assays for cobicistat (De Nicolò et al. 2020; Gouget et al. 2020; Penchala et al. 2016; Simiele et al. 2017) and venetoclax (Eisenmann et al. 2020; El‐Gendy et al. 2025; Gao et al. 2023; Reddy et al. 2022; X. Yang et al. 2022; Y. Yang et al. 2023) have been described in several matrices. However, for PK boosting of venetoclax, cobicistat needs to be quantified simultaneously with venetoclax in human plasma and serum. Therefore, the objective of this study was to develop and validate a rapid LC–MS/MS analytical method for the simultaneous determination of cobicistat and venetoclax in plasma and serum.

## 2 Experimental

### 2.1 Chemicals

The following reagents with corresponding suppliers were used in the experiments: cobicistat, [^13^C_4_,^2^H_3_] cobicistat, venetoclax, [^2^H_7_] venetoclax, (Alsachim, France), dimethyl sulfoxide (DMSO) (Merck, Netherlands), acetonitrile, methanol (Biosolve, Netherlands), ammonium formate (Thermo Scientific, USA), blank human EDTA plasma (BioIVT, USA), and blank serum (Merck Millipore).

### 2.2 Chromatography

**FIGURE 1:**
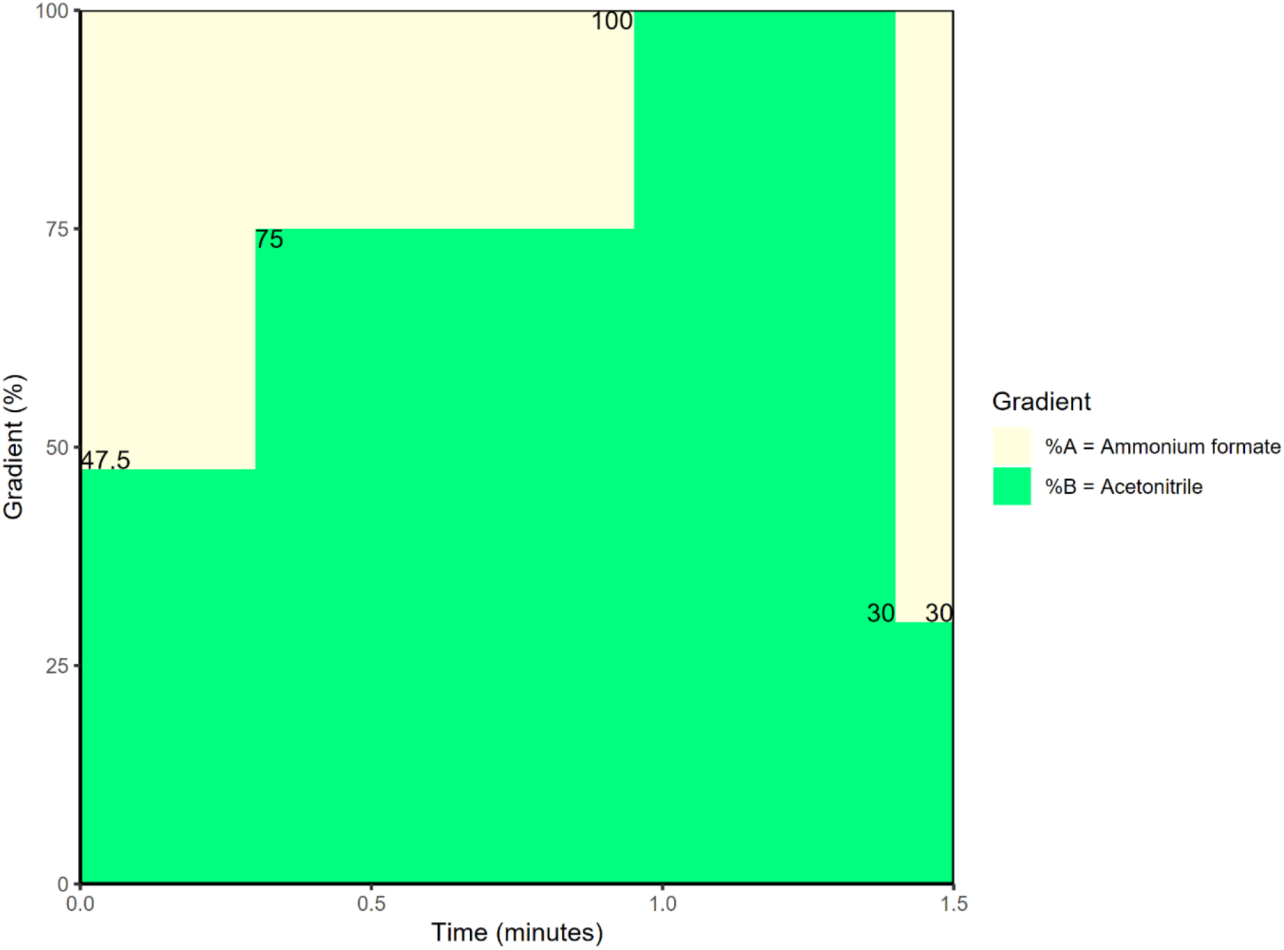
Elution process. Percentages (%) mobile phase versus time (minutes).

Chromatographic separation was performed using an ultra‐high‐performance liquid chromatography (UHPLC) system with a C18 column (2.6 μm, 50 × 2.1 mm, Accucore, Thermo Fisher Scientific, USA). The LC dwell volume of the system was approximately 350 μL. The autosampler and column oven temperatures were set at 10°C and 60°C, respectively. The injection volume was 0.5 μL, and the injection needle was washed with a methanol/water (4:1) mixture after each injection. The elution gradient involved two mobile phases: mobile phase A (20 mmol/L ammonium formate, buffered at pH 3.5 with formic acid), and mobile phase B (acetonitrile). The elution process was as follows: 0.00 min (30.0% B), 0.000–0.300 min (47.5% B), 0.300–0.950 min (75% B), 0.950–1.400 min (100% B), 1.400–1.500 min (30% B), 1.500 min (30.0% B). The flow rate was 1.000 mL/min. The elution process is depicted in Figure 1.

### 2.3 Mass Spectrometry

The mass spectrometry system included a TSQ Quantiva Triple Quadrupole, Vanquish Autosampler, Vanquish Horizon Binary Pump, Vanquish Column Compartment, and Vanquish Charger (Thermo Fisher Scientific, USA). A heated electrospray ionization (H‐ESI) ion source with positive polarity and a static spray voltage of 1500 V was used. The sheath, auxiliary, and sweep gas pressures were 60, 25, and 0 a.u., respectively. The ion transfer tube and vaporizer temperatures were set at 140°C and 350°C, respectively. Selected reaction monitoring (SRM) was used to detect the analytes and their internal standards (IS). The cycle time was 0.05 s, with Q1 and Q3 resolutions at 0.7 full width at half maximum (FWHM), and collision‐induced dissociation (CID) gas at 1.5 mTorr. SRM conditions for the analysis are detailed in Table 1. Raw LC‐MS data were processed with Thermo Scientific Xcalibur Version 4.4.16.14. Key mass fragments were based on previous LC‐MS/MS assays for cobicistat and venetoclax (Liu et al. 2017; Penchala et al. 2016).

**TABLE 1:** SRM conditions.

Analyte	Retention time (min)	RT window (min)	Precursor (*m*/*z*)	Product (*m*/*z*)	Collision energy (V)	Min dwell time (ms)
Cobicistat	0.45	0.4	776.4	545.3	36	22.707
Cobicistat [^13^C_4_,^2^H_3_]	0.45	0.4	783.4	552.3	36	22.707
Venetoclax	0.85	0.4	868.4	321.1	41	22.707
Venetoclax [^2^H_7_]	0.85	0.4	875.4	321.1	41	22.707

### 2.4 Stock and Working Solutions

Stable isotope‐labelled [^13^C_4_,^2^H_3_] cobicistat and [^2^H_7_] venetoclax were used as IS (Figure 3). Solutions of cobicistat and venetoclax were prepared in DMSO and were serially diluted in DMSO. These solutions were then spiked to serum to achieve the desired concentrations. DMSO final concentrations were < 5% for the dilution quality control (QC) levels and < 2.5% for the remaining QC levels. Calibration curves for cobicistat ranged from 5 to 500 μg/L (5, 10, 25, 50, 100, 200, 400, and 500 μg/L) and for venetoclax from 50 to 5000 μg/L (50, 100, 250, 500, 1000, 2000, 4000, and 5000 μg/L). QC samples consisted of lower limit of quantification (LLQ), low, medium, and high level. The final QC concentrations were 5, 10, 200, and 400 μg/L for cobicistat and 50, 100, 2000, and 4000 μg/L for venetoclax, for LLQ, low, medium, and high, respectively. The calibrators and QC were made from independent sets of stocks. Stock solutions of the internal standards were diluted in methanol to obtain working IS concentrations: [^13^C_4_,^2^H_3_] cobicistat at 20 μg/L and [^2^H_7_] venetoclax at 100 μg/L.

### 2.5 Sample Preparation

100 μL of serum or EDTA plasma was added to a 1.5‐mL screw vial (Fisher Scientific). Subsequently, 500 μL of IS was added, and the mixture was vortexed for 1 min. The mixture was then centrifuged for 5 min at 9500 g, and the vials were transferred to the autosampler for further analysis in the LC‐MS/MS system.

### 2.6 Method Validation

Method validation was conducted according to the harmonized ICH M10 guideline used by EMA and FDA (EMA 2022). Additionally, a matrix comparison between human serum and human plasma was performed.

#### 2.6.1 Selectivity

Selectivity was assessed by analyzing mass spectrometry signals in six independent blank serum samples. Interfering signals were compared with the response signal of the IS and the analyte at the LLQ. Interfering signals should not exceed 20% of the analyte signal at LLQ and should be less than 5% of the IS signal.

#### 2.6.2 Carryover

Carryover was assessed by injecting blank samples after the highest limit of quantification (HLQ) sample. Eluting peaks on the chromatogram of the blank serum samples were evaluated at the retention times of cobicistat and venetoclax. Acceptance criteria for carryover were that signals should not exceed 20% of the analyte signal at LLQ and should be less than 5% of the IS peak signal.

#### 2.6.3 Linearity Range

Linearity of the calibration curves was assessed by analyzing three independent runs on three different days. The peak height ratio of analyte/IS was plotted versus the concentration (x) with a weighting factor of 1/x. Back‐calculated concentrations based on calibrators LLQ and HLQ of all eight calibration points should be within ±15% of the nominal concentrations, except for LLQ, which should be within ±20%. At least 75% of the calibration points should meet these criteria in each run.

#### 2.6.4 Accuracy, Sensitivity, and Precision

Within‐day and between‐day accuracy and precision were determined for four QC concentration levels (LLQ, low, medium, and high) in five replicates on three different days. Sample accuracy and precision criteria should be within ±15% of the nominal values, whereas for LLQ samples, they should be within 20% of the nominal value.

#### 2.6.5 Dilution Effect

Dilution integrity was assessed by calculating the accuracy and precision for a diluted sample with an initial concentration higher than HLQ of 1000 and 10,000 μg/L for cobicistat and venetoclax, respectively. Samples were diluted in plasma with a 10× dilution factor to a concentration within the calibration curve range. The accuracy and precision of the diluted sample should be within ±15% of the nominal values.

#### 2.6.6 Matrix Effect and Recovery

The matrix effect and recovery were tested in six independent batches at three different concentration levels (low, medium, and high) in human serum according to the EMA guideline on bioanalytical method validation (EMA 2011). The matrix effect was calculated by dividing the peak height of spiked extracts of blank serum by the peak height of spiked precipitation solvent (i.e., methanol). The IS‐normalized matrix factor was calculated by dividing the peak height ratio (i.e., analyte/IS) of spiked extracts of blank serum by the peak height ratio of spiked precipitation solvent (i.e., methanol). The acceptance limit for the coefficient of variation (CV) of the IS‐normalized matrix factor of the six lots of human serum is 15%.

Recovery was calculated by dividing the peak height ratio (i.e., analyte/IS) of spiked samples in serum by the peak height ratio (i.e., analyte/IS) in spiked extracts of blank serum with the analytes after extraction. Recovery requirement was consistency and reproducibility regardless of the QC concentration level.

#### 2.6.7 Stability

All analytes were tested for stability according to EMA and FDA guidelines under various conditions, including autosampler, freeze–thaw cycles, refrigerator, and room temperature stability tests. Stability at room (20°C–25°C) and refrigerator (5°C) temperatures was assessed at two concentration levels over 8 days. The analyte peak height ratio was evaluated at different time points, and accuracy was assessed by calculating the deviation from the value at *t* = 0. Freeze–thaw tests were conducted at two concentration levels over three cycles. Autosampler stability was assessed for 7 days at 10°C. All stability tests were performed thrice in human serum for the low and high QC concentrations. According to EMA and FDA guidelines, the analyte is considered stable if the accuracy does not exceed ±15%.

#### 2.6.8 Matrix Comparison

In plasma, accuracy and precision for four QCs (LLQ, low, medium, and high) per analyte were calculated based on calibrators LLQ and HLQ derived from human serum samples for five replicates. Matrix comparison was accepted if accuracy was within ±15% (20% for LLQ) of the nominal concentrations and if precision was less than 15% (20% for LLQ).

### 2.7 Clinical Application

The assay has been implemented for TDM of venetoclax and cobicistat in our hospital. TDM of cobicistat is primarily used as a measure of adherence. TDM of venetoclax is used in the management of drug–drug interactions, due to its extensive metabolism by CYP3A4.

## 3 Results

### 3.1 Selectivity

**FIGURE 2:**
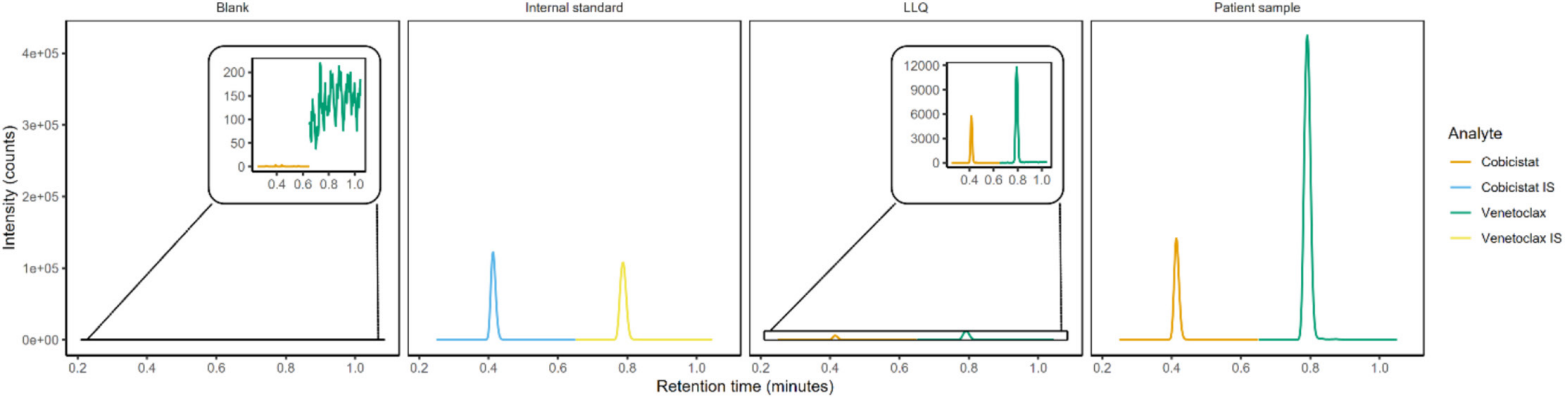
Chromatograms of blank human serum, internal standard, LLQ in human serum, and a patient sample for cobicistat and venetoclax. The insets in the blank and LLQ chromatograms are magnifications.

**FIGURE 3:**
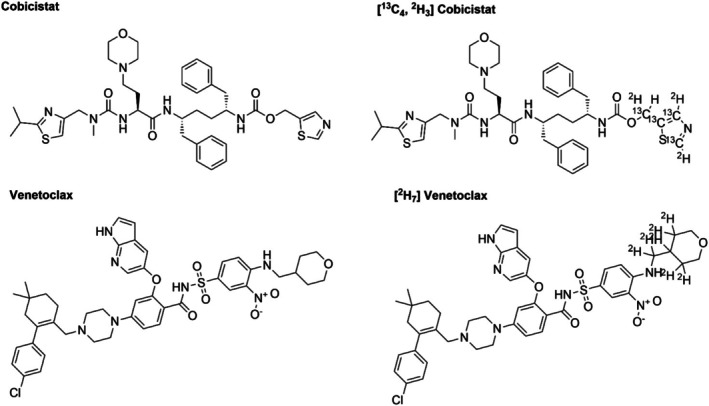
Chemical structure of cobicistat, [^13^C_4_,^2^H_3_] cobicistat, venetoclax, and [^2^H_7_] venetoclax.

Interfering signal peak heights were all less than 20% of the analyte peak height and less than 5% of the internal standard (IS) peak height for both LLQ levels of cobicistat and venetoclax. This indicates that the method distinguishes the analytes from other components in the sample matrix. Figure 2 shows the chromatograms of the assay for blank, LLQ, and a patient sample for cobicistat and venetoclax, respectively. Figure 3 shows the chemical structure of cobicistat, [^13^C_4_,^2^H_3_] cobicistat, venetoclax, and [^2^H_7_] venetoclax. The method complied with EMA and FDA criteria for selectivity, ensuring reliable detection and quantification of cobicistat and venetoclax in serum samples.

### 3.2 Carryover

Carryover effects in blank serum samples were all less than 20% of the analyte peak height at LLQ and less than 5% of the IS peak height after injection of the HLQ levels of cobicistat and venetoclax. This demonstrates that the method minimizes residual analyte signals between injections. Thus, the method met EMA and FDA criteria for carryover, ensuring reproducible results.

### 3.3 Linearity Range

The calibration curve included eight concentrations ranging from 5 to 500 μg/L (5, 10, 25, 50, 100, 200, 400, and 500 μg/L) for cobicistat and 50 to 5000 μg/L (50, 100, 250, 500, 1000, 2000, 4000, and 5000 μg/L) for venetoclax. The accuracy of back‐calculated concentrations ranged from −8.0% to 2.9% of all eight calibration points for cobicistat (Table 2). The accuracy of back‐calculated concentrations ranged from −7.2% to 4.6% of all eight calibration points for venetoclax (Table 2). The correlation coefficient (*r* ^2^) was 1.00 for cobicistat and venetoclax. All calibration points were within ±15% of the nominal concentrations, indicating a linear relationship. This complied with EMA and FDA criteria for linearity, and this method is therefore able to produce consistent and reliable quantification across a wide range of concentrations.

**TABLE 2:** Accuracy of calibration ranges for cobicistat and venetoclax.

Analyte	Nominal concentration calibrator (μg/L)	Accuracy (%)		
		Day 1	Day 2	Day 3
Cobicistat	5	1.1	−0.9	−0.3
	10	0.4	−2.7	−5.2
	25	−2.9	−3.2	−7.7
	50	−8.0	−1.3	−5.0
	100	−7.2	0.0	−3.5
	200	−3.5	0.9	−0.1
	400	−1.3	2.9	1.3
	500	−0.6	−0.1	1.6
Venetoclax	50	1.2	−0.7	1.6
	100	−3.5	−0.9	−3.4
	250	−5.2	−3.4	−6.2
	500	−7.2	−1.3	−4.6
	1000	−4.9	0.1	−3.2
	2000	−2.0	1.3	−0.1
	4000	−0.7	4.6	1.3
	5000	−0.6	0.1	1.3

### 3.4 Accuracy and Precision

Accuracy ranged from −5.9% to 2.4%, within‐day precision ranged from 1.2% to 4.8%, and between‐day precision ranged from 0.4% to 4.3%, as shown in Table 3. The low accuracy and precision values indicate that the method consistently produces results close to the nominal values. Therefore, the method adhered to EMA and FDA criteria for accuracy and precision.

**TABLE 3:** Accuracy and precision.

Analyte	QC level	Nominal concentration (μg/L)	Accuracy (%)				Precision (CV %)	
			Day 1 (*n* = 5)	Day 2 (*n* = 5)	Day 3 (*n* = 5)	Average	Within‐day (*n* = 15)	Between‐day (*n* = 15)
Cobicistat	LLQ	5	−5.2	−0.2	−2.7	−2.7	4.8	1.5
	Low	10	−7.6	−2.7	−3.7	−4.7	3.5	2.2
	Medium	200	−6.8	−1.7	−4.4	−4.3	1.2	2.6
	High	400	−3.5	3.3	−0.8	−0.3	1.6	3.4
	Dilution	1000	−9.1	−2.8	−5.9	−5.9	1.4	3.3
Venetoclax	LLQ	50	−5.6	3.5	−0.1	−0.8	3.6	4.3
	Low	100	−5.1	−1.9	−1.0	−2.7	1.6	2.1
	Medium	2000	−0.3	0.4	−1.6	−0.5	2.1	0.4
	High	4000	1.4	4.6	1.1	2.4	1.4	1.8
	Dilution	10,000	−3.6	−1.4	−2.5	−2.5	2.0	0.7

### 3.5 Matrix Effect and Recovery

Matrix effect ranged from −9.3% to −2.8%, IS normalized matrix effect ranged from −3.0% to 2.8%, and recovery ranged from 101.8% to 105.5%, as shown in Table 4. The method adhered to EMA and FDA criteria for matrix effect.

**TABLE 4:** Matrix effect and recovery.

Analyte	QC level	Matrix effect (%)	Matrix effect (CV %)	Matrix effect IS normalized (%)	Matrix effect IS normalized (CV %)	Recovery (%)
Cobicistat	Low	−2.8	6.7	−2.1	6.9	103.7
	Medium	−3.3	3.7	−3.0	3.3	105.5
	High	−4.2	1.3	−2.8	2.0	105.0
Venetoclax	Low	−4.8	5.5	2.8	3.8	101.8
	Medium	−9.3	10.9	−0.9	4.0	104.0
	High	−7.8	7.4	−1.1	3.2	104.6

### 3.6 Stability

Stability data for cobicistat and venetoclax are presented in Table 5. Both drugs were stable for at least 8 days when stored at room temperature and refrigerated. Autosampler stability was maintained for at least 10 days for both cobicistat and venetoclax. Additionally, both drugs remained stable through three freeze–thaw cycles. These stability results ensure that the analytes remain stable and quantifiable under various storage and handling conditions.

**TABLE 5:** Stability.

		Room temperature (8 days) (*n* = 3)		Refrigerator (8 days) (*n* = 3)		Autosampler (12 days) (*n* = 3)		Freeze–thaw (3 cycles) (*n* = 3)	
Analyte	QC level	Accuracy (%)	Precision (CV %)	Accuracy (%)	Precision (CV %)	Accuracy (%)	Precision (CV %)	Accuracy (%)	Precision (CV %)
Cobicistat	Low (10 μg/L)	−1.0	1.5	0.5	1.9	2.4	0.8	−1.1	3.3
	High (400 μg/L)	−4.5	1.5	0.1	1.7	5.0	2.0	−2.1	1.1
Venetoclax	Low (100 μg/L)	0.2	0.6	1.2	0.8	0.0	1.5	−2.1	3.3
	High (4000 μg/L)	−3.7	0.7	−0.5	0.5	−0.7	1.5	−3.3	1.2

### 3.7 Matrix Comparison

Accuracy ranged from −2.9% to 3.4%, precision ranged from 0.6% to 4.3%, for cobicistat in plasma. Accuracy ranged from −5.7% to −0.1%, precision ranged from 0.9% to 2.9%, for venetoclax in plasma. The data are depicted in Table 6. Therefore, this assay can be used to determine cobicistat and venetoclax in plasma and serum.

**TABLE 6:** Matrix comparison in plasma.

Analyte	QC level	Accuracy (%) (*n* = 5)	Precision (CV %) (*n* = 5)
Cobicistat	LLQ	−1.3	4.3
	Low	−2.9	1.6
	Medium	1.0	1.0
	High	3.4	0.6
Venetoclax	LLQ	−4.5	2.9
	Low	−5.7	2.2
	Medium	−1.4	2.8
	High	−0.1	0.9

### 3.8 Clinical Application

Between February 2023 and January 2025, 13 cobicistat samples and 17 venetoclax samples were analyzed for routine TDM purposes. The mean cobicistat concentration ± standard deviation (SD) was 138.8 ± 123.3 μg/L, and the mean venetoclax concentration ± SD was 1497.1 ± 1285.9 μg/L.

## 4 Conclusion

In this study, we developed and validated a rapid LC‐MS/MS assay for the simultaneous quantification of cobicistat and venetoclax in plasma and serum samples. The method met the criteria set by the EMA and FDA, ensuring its reliability and accuracy. With a runtime of 1.6 min, the assay is well suited for the rapid analysis of urgent samples in TDM and the efficient processing of large quantities in pharmacokinetic studies.

## Data Availability

The data that support the findings of this study are available from the corresponding author upon reasonable request.
